# One-pot synthesis of carbon supported calcined-Mg/Al layered double hydroxides for antibiotic removal by slow pyrolysis of biomass waste

**DOI:** 10.1038/srep39691

**Published:** 2016-12-21

**Authors:** Xiaofei Tan, Shaobo Liu, Yunguo Liu, Yanling Gu, Guangming Zeng, Xiaoxi Cai, ZhiLi Yan, Chunping Yang, Xinjiang Hu, Bo Chen

**Affiliations:** 1 College of Environmental Science and Engineering, Hunan University, Changsha, 410082, P.R. China; 2 Key Laboratory of Environmental Biology and Pollution Control (Hunan University), Ministry of Education, Changsha, 410082, P.R. China; 3 School of Metallurgy and Environment, Central South University, Changsha, 410083, PR China; 4 School of Architecture and Art, Central South University, Changsha, 410083, PR China; 5 College of Environmental Science and Engineering Research, Central South University of Forestry and Technology, Changsha, 410004, PR China

## Abstract

A biochar supported calcined-Mg/Al layered double hydroxides composite (CLDHs/BC) was synthesized by a one-pot slow pyrolysis of LDHs preloaded bagasse biomass. Multiple characterizations of the product illustrated that the calcined-Mg/Al layered double hydroxides (CLDHs) were successfully coated onto the biochar in slow pyrolysis of pre-treated biomass. The as-synthesized CLDHs/BC could efficiently remove antibiotic tetracycline from aqueous solutions. The coating of CLDHs significantly increased the adsorption ability of biochar, and CLDHs/BC exhibited more than 2 times higher adsorption capacity than that of the pristine biochar (BC) in the tested pH range. The maximum adsorption capacity of CLDHs/BC for tetracycline was 1118.12 mg/g at 318 K. The experimental results suggested that the interaction with LDHs on biochar played a dominant role in tetracycline adsorption, accompanied with π–π interaction and hydrogen bond. This study provides a feasible and simple approach for the preparation of high-performance material for antibiotics contaminated wastewater treatment in a cost-effective way.

Recently, non-regulated trace organic emerging contaminants (ECs) including halogenated flame retardants, surfactants, pharmaceuticals, illicit drugs and personal care products have caused increasing public concerns^1^,^2^,^3^,^4^. As one of pharmaceuticals, antibiotics has been commonly used in the world to resist disease and prevent humans and animals from microbial infections^5^. The extensive use of antibiotics resulted in frequent detection of their residues in final effluents of wastewater treatment plants around the world^6^,^7^,^8^. Therefore, it is imperative to tackle the antibiotics entering wastewater before discharging into the aquatic environment.

Layered double hydroxides (LDHs) are kinds of lamellar inorganic materials which can be described by the general formula [M_1_–_*x*_^2+^M_*x*_^3+^(OH)_2_]^*x*+^(A^n–^)_*x*/n_·*m*H_2_O. M^2+^ and M^3+^ are divalent (e.g., Mg^2+^, Co^2+^, Ni^2+^, Zn^2+^, Cu^2+^) and trivalent cations (e.g., Al^3+^, Fe^3+^, Ga^3+^), respectively; A^n–^ is interlayer gallery anion (e.g., CO_3_^2−^, Cl^−^, NO_3_^−^, SO_4_^2−^); and *x* is the molar ratio of M^3+^/(M^2+^ + M^3+^) and the layer charge will depend on the M^2+^/M^3+^ ratio^9^,^10^,^11^. By heating to 450–500 °C, LDHs can be converted into mixed metal oxides (MMOs), which exhibit fine dispersion of metal cations and high surface area. An important property of MMOs is the so-called “memory effect”, that is the calcined anionic clays can reconstruct their original layered structure after adsorption of various anions^12^. LDHs and CLDHs have been widely applied as adsorbents to remove pharmaceuticals from aqueous solutions in recent years^13^,^14^,^15^. LDHs and CLDHs particles can also be loaded onto high surface area carbonaceous materials to further improve the dispersion of the LDHs and CLDHs particles, thus improving the performance and reducing the cost of LDHs and CLDHs.

Biochar is a carbon-rich solid derived by pyrolyzing biomass with little or no oxygen^16^, which is usually produced from crop residues, wood biomass, animal litters, and solid wastes via various thermochemical processes^17^. The resultant biochar usually exhibit high porosity, enriched surface functional groups and mineral components due to the removal of the moisture and the volatile matter contents of the biomass by thermal treatment, which has been widely applied as a sorbent for contaminant management in soil and water^18^,^19^,^20^,^21^. Therefore, biochar can serve as a carrier material for the LDHs and CLDHs, which helps to minimize the pulverization of hydrotalcite^22^, and in turn, LDHs and CLDHs can also functionalize biochar materials for wastewater treatment^23^. Compared with other carrier materials, multiple advantages of using biochar as the substrates material are existed, including its abundant and low-cost feedstocks (agricultural biomass and solid waste), lower energy requirements during production, and concomitant energy production (biofuels and syngas)^21^,^24^. Studies have also reported that biochar can efficiently remove antibiotics from aqueous solutions^25^,^26^. Considering the respective superior properties of LDHs and biochar, the synthesis of biochar supported nano-Mg/Al layered double hydroxides might be a potent method for expanding both the application of LDHs and biochar in antibiotics treatment.

The slow pyrolysis of biomass wastes has been demonstrated to be a promising technology that can produce renewable fuels and massive biochar products with a stable carbon skeleton^27^. In addition, LDHs can be converted into mixed metal oxides (MMOs) by heating to 450–500 °C during pyrolysis process^11^. Therefore, it is possible to obtain a highly active carbon supported CLDHs using the one-pot slow pyrolysis reaction of LDHs pre-coated biomass. During the pyrolysis process, pyrolysis played dual role for both converting biomass into biochar and calcining nanosized LDHs simultaneously, which could cut the production cost and time. In the present study, we focused our efforts on the following: (1) synthesis of a carbon supported calcined-Mg/Al layered double hydroxides composite (CLDHs/BC) by the one-pot slow pyrolysis of LDHs preloaded bagasse biomass, (2) characterization of the resulting CLDHs/BC using a variety of analytical techniques, (3) demonstration of the adsorption ability of the prepared CLDHs/BC for antibiotic tetracycline, and (4) investigation of the underlying mechanisms of tetracycline adsorption onto CLDHs/BC. This study provides a feasible and simple approach for the preparation of high-performance material for antibiotics contaminated effluents treatment in a cost-effective way.

## Results and Discussion

### Characterization of CLDHs/BC

**Figure 1:**
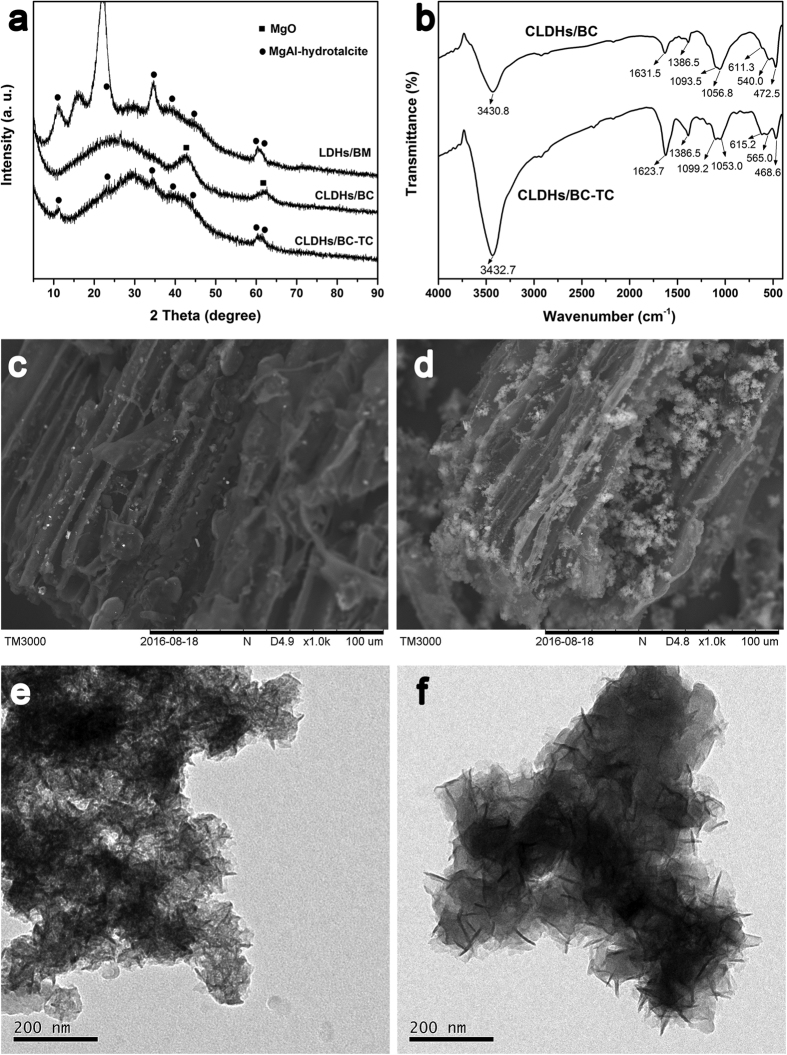
(**a**) The XRD pattern of Mg/Al LDHs pre-coated bagasse biomass (LDHs/BM), biochar supported calcined-Mg/Al layered double hydroxides composite (CLDHs/BC) and CLDHs/BC loaded with TC (CLDHs/BC-TC); (**b**) The FTIR spectra of CLDHs/BC and CLDHs/BC-TC; The SEM images of (**c**) pristine biochar (BC), and (**d**) CLDHs/BC; The TEM images of (**e**) CLDHs/BC, and (**f**) CLDHs/BC-TC.

The XRD pattern in the range of 5–85° of the Mg/Al LDHs pre-coated bagasse biomass (LDHs/BM) sample is shown in Fig. 1a. The XRD patterns exhibit the characteristic diffractions of hydrotalcite (indexed by the JCPDS X-ray powder diffraction file of No. 22–700.), indicating that the Mg/Al-hydrotalcite was successfully coated onto the bagasse biomass by liquid-phase deposition method. Some unidentified peaks emerged in the XRD pattern, suggesting the presence of other minerals, which are existed in the bagasse biomass. The XRD patterns of CLDHs/BC showed the disappearance of the peaks of hydrotalcite (Fig. 1a) and the appearance of broad peaks attributed to the formation of Mg/Al mixed oxides. During the pyrolysis of bagasse biomass at 475 °C, hydrotalcite was calcined and resulted in an almost complete collapse of the structure of Mg/Al-LDHs by the decomposition of the hydrotalcite-CO_3_^2−^, evolving CO_2_ and water^28^. Consequently, Mg/Al oxide was formed, which is capable of being restored to hydrotalcite when rehydrated.

The FTIR spectra of CLDHs/BC before and after adsorption of TC are presented in Fig. 1b. The intense band at 3430.8 cm^−1^ was ascribed to the O−H stretching vibrations of hydrogen-bonded hydroxyl groups. The band at 1631.5 cm^−1^ was corresponding to C=C stretching vibrations. The intense band at 1386.5 cm^−1^ was assigned to the bending vibrations of –CH_3_ groups. The band at 1056.8 cm^−1^ was assigned to the C–O stretching vibrations. A series of bands recorded in the 400–800 cm^–1^ region were ascribed to the M–O and O–M–O (M = Mg, Al) vibration. The FTIR spectra confirmed that the Mg/Al oxides converted from Mg/Al-hydrotalcite was presented in the biochar composites.

The SEM images of BC and CLDHs/BC are shown in Fig. 1. As can be seen, both BC and CLDHs/BC exhibited irregular surfaces with pores of different shapes and sizes. Compared with BC, the SEM images of CLDHs/BC revealed that the CLDHs were deposited on the biochar surfaces evenly (Fig. 1d), which confirmed that the nanosized CLDHs particles were successfully coated on the biochar surface. The TEM image (Fig. 1e) revealed that the calcined MgAl-LDHs on biochar had collapsed layer structure with no obvious hexagonal dimension, which further indicated that MgAl-LDHs was converted to magnesium and aluminum oxides after pyrolysis.

### The effect of adsorbent dosage on TC adsorption

The effects of the CLDHs/BC dosage on the adsorption of TC was studied. As can be seen from Fig. S1, the removal percentage of TC increased with increasing dosage of CLDHs/BC. However, the adsorption capacity of TC decreased with the increasing adsorbent dose. This was mainly attributed to that more bonding sites were available for TC with the increase of CLDHs/BC dosage, resulting in the increase of removal rate. Further high adsorbent dosage provided excess amount of the active sites leading to a lower utility of the sites at a certain concentration of TC solution^29^.

### The effect of ionic strength on the adsorption capacity

**Figure 2:**
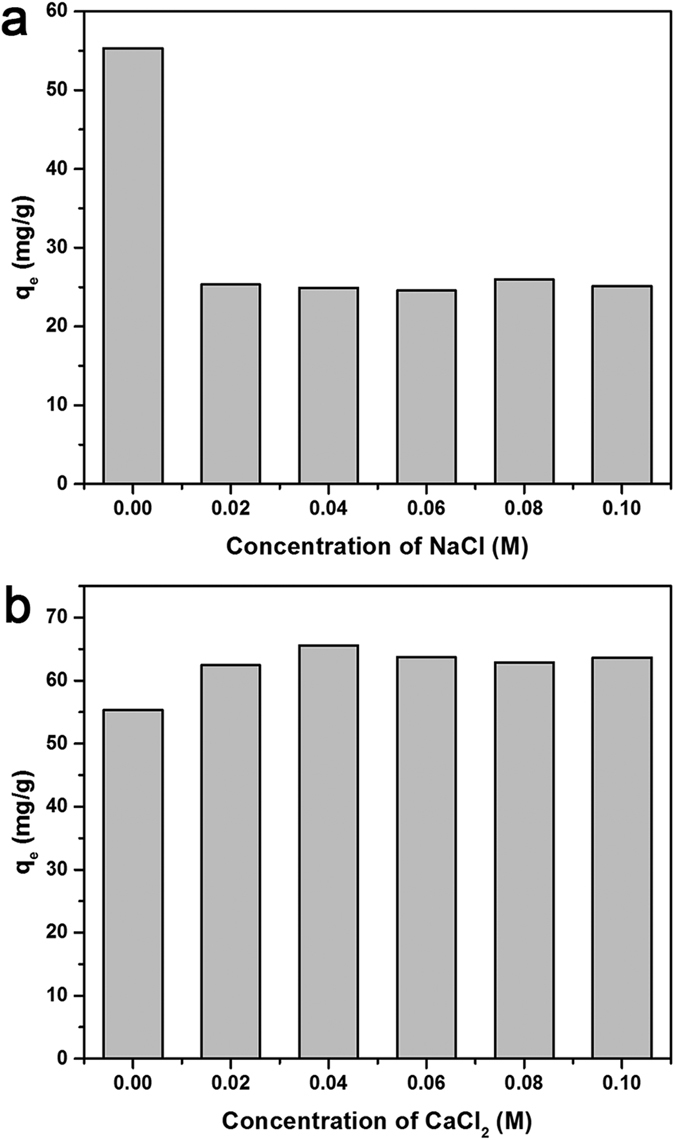
Effects of the ionic strength on the adsorption of TC by CLDHs/BC: (**a**) effects of NaCl and (**b**) effects of CaCl_2_.

As the actual wastewater usually consist of high concentration of salts, which may affect the TC adsorption. Therefore, the influence of salt ionic strength on the removal of TC by CLDHs/BC was studied with the sodium chloride and calcium chloride concentration varying from 0 to 0.1 M. The effects of sodium chloride and calcium chloride on the TC adsorption by CLDHs/BC are shown in Fig. 2. It can be seen that the adsorption capacities decreased with the addition of NaCl (Fig. 2a). However, the nagative effect changed little among different Na^+^ concentrations. The same inhibition trends were also observed by Gao, *et al*.^30^. When the initial concentration of tetracycline was 50 mg/L, the adsorption capacities decreased by more than 50% after the addition of various concentrations of NaCl. This can be because that NaCl may influence adsorption capacities of tetracycline on CLDHs/BC by the electrostatics screening effect on the electrostatic interaction between the TC cations and CLDHs/BC groups^30^. And, Na^+^ may occupy the actives sites, which are available for TC bonding. However, CaCl_2_ slightly increased the adsorption capacity and remained almost at the same level with increasing concentration of CaCl_2_ (Fig. 2b). This may be attributed to the formation of complexation of tetracycline with Ca^2+^ ions^31^.

### Effect of solution pH on TC adsorption

**Figure 3:**
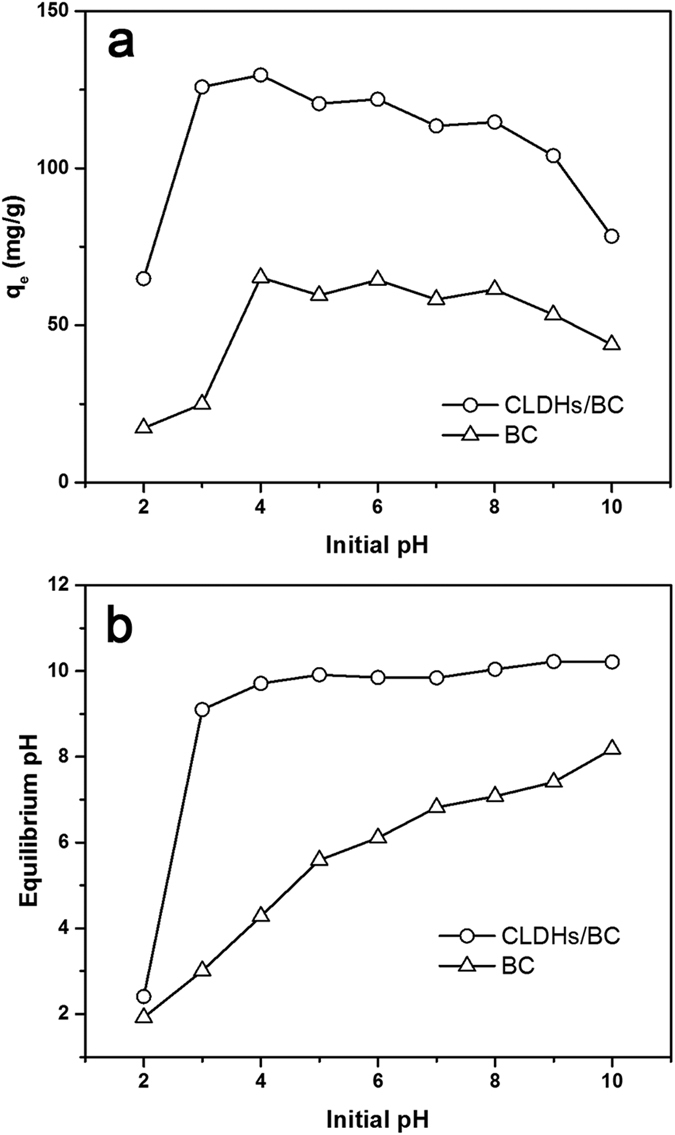
(**a**) Effect of the initial pH on the adsorption of TC by CLDHs/BC and BC. (**b**) Relationship between the initial and equilibrium pH of the sample solution.

The initial solution pH is one of the most vital parameters for the TC removal, because it affects the surface charge of adsorbent, the ionic state of functional groups on the adsorbent surface as well as the form of TC species in the adsorption system. The effects of pH on the adsorption properties of TC by CLDHs/BC and BC were determined. As shown in Fig. 3a, the adsorption amount of TC by three adsorbents all increased with the increase of pH from 2.0 to 4.0. However, when the pH were higher than 4.0, the adsorption capacity changed little with a slight decrease with increasing of pH. The results indicated that CLDHs/BC and BC reached their maximum adsorption capacity at pH around 4.0. It could be seen that the adsorption behavior of TC by these adsorbents showed clearly dependence on solution pH. This might be ascribed to the molecular structural characteristics of TC and various active sits on the surface of adsorbents. In addition, CLDHs/BC exhibited more than 2 times higher adsorption capacity than that of the pristine biochar (BC) in the tested pH range, suggesting that the coating of CLDHs significantly increased the adsorption ability of biochar.

The initial and equilibrium pH of the sample solution are shown in Fig. 3b. As can be seen, the equilibrium pH of BC solution increased gradually with the increase of initial pH. From the initial pH = 2 to pH > 2, the equilibrium pH CLDHs/BC significantly increased from 2.41 to 9.10-10.22. In addition, when the initial pH was higher than 2, the equilibrium pH of CLDHs/BC changed little with slight increase. Under different acid dissociation constants (p*K*_a_ = 3.3, 7.7, and 9.7), TC exists as cationic (TCH_3_^+^), zwitterionic (TCH_2_^±^), and anionic (TCH^−^ or TC^2−^) species (Figs S2 and 3). Under the lower initial pH, the surface of adsorbents were positively charged and TC mainly existed as TCH_3_^+^, resulted in the electrostatic repulsion between the positively charged surface and the cationic TC. When pH > 2, the significant increase of solution pH suggested that TC existed as anionic (TCH^−^ or TC^2−^) species and the surface of adsorbents were negatively charged, which also resulted in the electrostatic repulsion. Therefore, electrostatic attraction could not deduced to be the main mechanism of TC adsorption onto CLDHs/BC. Other mechanisms may be account for the initial rapid increase of adsorption and the further high adsorption ability of CLDHs/BC.

### Adsorption kinetics

**Figure 4:**
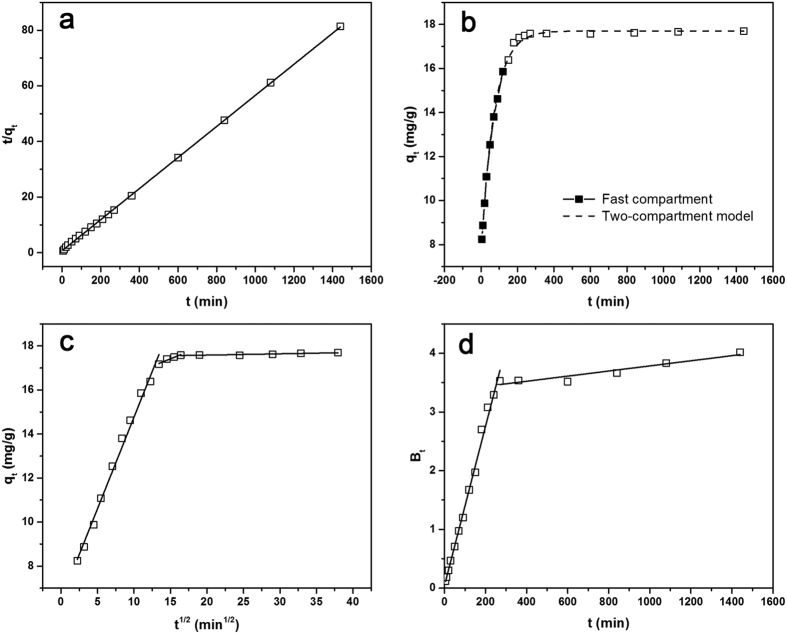
The kinetics for TC adsorbed by CLDHs/BC: (**a**) Modeled result for TC sorption using the pseudo-second-order equation; (**b**) Two-compartment model for the adsorption of TC onto the CLDHs/BC (**c**) Intraparticle diffusion plots of adsorption capacity *q*_t_ versus the square root of time *t*^0.5^ for the adsorption of TC onto the CLDHs/BC; (**d**) Plots of Boyd parameter *B*_t_ versus time t for the adsorption of TC onto the CLDHs/BC.

In order to determine the equilibration time for adsorption of TC and to investigate the kinetics of adsorption process, the adsorption of TC on CLDHs/BC as a function of contact time was studied. The pseudo-first-order and pseudo-second-order models were applied to simulate the experimental kinetic data^32^,^33^,^34^. Detailed information of those models is described in the Supplementary data. The results of adsorption kinetics studies are shown in Fig. 4 and the values of parameters are summarized in Table 1. The data showed a better fit to pseudo-second-order model (*R*^2^ = 0.999) than pseudo-first-order model (*R*^2^ = 0.702), which can be further confirmed by the excellent close between the calculated *q*_e_ value from pseudo-second-order model (*q*_e,cal_ = 17.90 mg/g) and the experimental results (*q*_e,exp_ = 17.70 mg/g).

Furthermore, the time-courses of TC sorption onto CLDHs/BC were analyzed with a two-compartment and first-order dynamics model (presented in the Supplementary data)^35^. This kinetics model considered the adsorption of TC by CLDHs/BC as a two-domain process, which could be divided into “fast” and “slow” compartments. As shown in Table 1, the two-compartment model well fitted the dynamics data of TC adsorption on CLDHs/BC with *R*^2^ = 0.998. The *F*_fast_ value of TC adsorption on CLDHs/BC were much greater than that of *F*_slow_, indicating that the fast sorption stage was predominant during TC sorption process. As shown in Fig. 4b, the fast compartment achieved 89.56% of its own sorption capacity of CLDHs/BC in a relatively short contact time of 120 min, while the sorption capacity contributed by slow compartment only reached about 10% between 120 min to 1440 min. The *K*_fast_ value of TC sorption is far higher than that of *K*_slow_, suggesting the relatively active reaction between TC and the adsorption sites of CLDHs/BC. The fast adsorption of TC by CLDHs/BC may be attributed to the interactions between TC species and the active sites including hydrotalcite and the functional groups on CLDHs/BC.

The intra-particle diffusion model was further examined to determine the diffusion mechanisms and identify the possible rate controlling procedure, which was an empirically functional relationship of adsorption amount at interval *t (q*_t_) with *t*^1/2^. As shown in Fig. 4c, the plots of *q*_t_ against *t*^1/2^ were multi-linear including three linear portions, indicating that multiple steps were involved in the adsorption process. The first section of the curve with a large slope corresponds to transport of TC from the bulk solution to the external surface of CLDHs/BC by film diffusion. The second section describes the gradual adsorption stage, corresponding to the diffusion of the TC molecules from the external surface into the pores of the CLDHs/BC (intra-particle diffusion). The third section with a small slope indicates the final equilibrium stage where the intra-particle diffusion starts to slow down^29^. The model parameters obtained from the sections of plots are listed in Table S2. As shown in Table S2, the values of *c*_i_ for all linear sections were not zero, which indicated that the intra-particle diffusion participated in the diffusion process but it was not the rate limiting step for the whole reaction^29^,^36^. During the adsorption process, TC molecules were initially adsorbed by the exterior surface of the CLDHs/BC, that the adsorption of TC onto CLDHs/BC was firstly controlled by film diffusion. Then, the sorption process was controlled by intra-particle diffusion as TC molecules further entered the pores of CLDHs/BC and were subsequently adsorbed by the interior surfaces.

To gain insights into the actual rate-controlling step involved in the overall TC sorption process, the adsorption kinetic data were further analyzed using the Boyd kinetic model^37^. Based on the analysis of the plot of this model, the actual rate-controlling step (film diffusion or intraparticle diffusion) involved in the overall TC sorption process can be decided. The plots of Boyd is shown in Fig. 4d and the model parameters obtained from the sections of plots are listed in Table S3. As can be shown, the plot of *B*_t_ versus *t* for the adsorption of TC onto CLDHs/BC was a straight line and did not pass through the origin, which suggested that film diffusion was the rate-controlling step in the initial adsorption process, and other mechanisms (intra-particle diffusion) took over subsequently^29^,^36^.

### Adsorption isotherms

**Figure 5:**
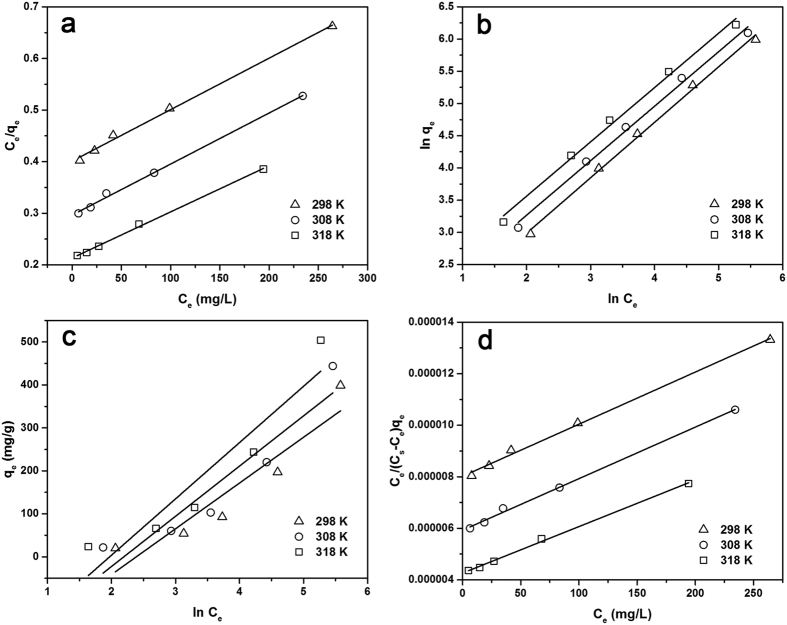
The equilibrium isotherms for TC adsorbed by CLDHs/BC at different temperatures: (**a**) the Langmuir model; (**b**) the Freundlich model; (**c**) The Temkin model; (**d**) The BET model.

The adsorption equilibrium isotherms was studied using four adsorption isothermal models including Langmuir, Freundlich, Tempkin and BET models (described in the Supplementary data) to fit the experimental data^32^,^33^,^34^. The Langmuir model assumes that a monomolecular layer is formed when adsorption takes place without any interaction between the adsorbed molecules. A linearity between *C*_e_/*q*_e_ and *C*_e_ was obtained and shown in Fig. 5. The values of Langmuir constants *q*_max_ and *K*_L_ are shown in Table 2. The experimental data exhibited high correlation with Langmuir model with the correlation coefficients *R*_2_ higher than 0.996 within the studied three temperatures, indicating the adsorption of TC onto the CLDHs/BC surface was probably a homogeneous and monolayer adsorption process^38^. Both *q*_max_ and *K*_L_ increased with increasing temperature, indicating the bonding between TC and active sites of CLDHs/BC was strengthened at higher temperature and the adsorption process was endothermic. Based on the Langmuir model, the maximum adsorption capacity of CLDHs/BC for TC was 1118.12 mg/g at 318 K. In addition, the *K*_L_ values in this study were calculated in the range from 0.043 to 0.128 L/mg, indicating that the adsorption between dye molecules and adsorbent was favorable (0 < *K*_L_ < 1).

The Freundlich isotherm is an empirical equation assuming that the adsorption process takes place on heterogeneous surfaces and adsorption capacity is related to the concentration of TC at equilibrium. The high correlation coefficients (*R*_2_ ≥ 0.992) for all temperatures tested indicated that the adsorption of TC onto the CLDHs/BC was in compliance with the Freundlich isotherm. The 1/*n* values are far less than 1, implying that the adsorption of TC onto CLDHs/BC was favorable at all temperatures studied. The increase of Freundlich constants (*K*_F_) with increase of temperature indicated that high temperature favored adsorption and the adsorption was endothermic in nature. However, the plots of Tempkin deviated from linearity at all the temperatures with low correlation coefficients (*R*^2^) ranged from 0.842 to 0.853. As an improvement on the Langmuir model, the BET adsorption model was based on the assumption that adsorbate could be adsorbed onto the adsorbent surface forming multilayer in a random distribution of adsorbed particle^39^. The high correlation coefficients (*R*_2_ ≥ 0.996) indicated that the BET model can also fit TC adsorption on CLDHs/BC preferably.

As can be seen, the adsorption data was fitted well to almost all the isotherm models, except the Tempkin equation. Fittings of the Langmuir, Freundlich and BET (*R*_2_ ≥ 0.992) matched the experimental data well, suggesting that the interaction between TC and the CLDHs/BC could be affected by both the Langmuir and the Freundlich processes. This result is consistent with the kinetics study results that the sorption of TC on the CLDHs/BC could be govern by multiple mechanisms.

### Thermodynamic analysis

Thermodynamic analysis was taken to gain further insights into sorption process and mechanisms, and it was investigated at three different temperatures (299, 309 and 319 K). The thermodynamic data, such as Gibbs free energy Δ*G*^0^, enthalpy Δ*H*^0^, entropy Δ*S*^0^, can be calculated using the relevant equations (described in the Supplementary data)^38^. The linear plot of ln *K*° versus 1/*T* for the adsorption of TC on CLDHs/BC is shown in Fig. S4. The calculated results are given in Table S4. The standard enthalpy and entropy changes of adsorption determined from Fig. S4 were 20.86 kJ/mol and 69.13 J/mol K, respectively, with a correlation coefficient of 0.992. The negative value of ∆*G*^0^ at three temperatures suggested the feasibility and spontaneous nature of the TC adsorption onto CLDHs/BC. Furthermore, the ∆*G*^0^ values decreased from −2.23 to −4.03 kJ/mol with the increase of temperature, indicating that the adsorption was more favorable at high temperature. The positive value of ∆*H*^0^ verified the endothermic nature of the adsorption process. The positive value of ∆*S*^0^ might be attributed to the increase of randomness at the solid-solution interface during the adsorption process.

### Adsorption mechanisms

**Figure 6:**
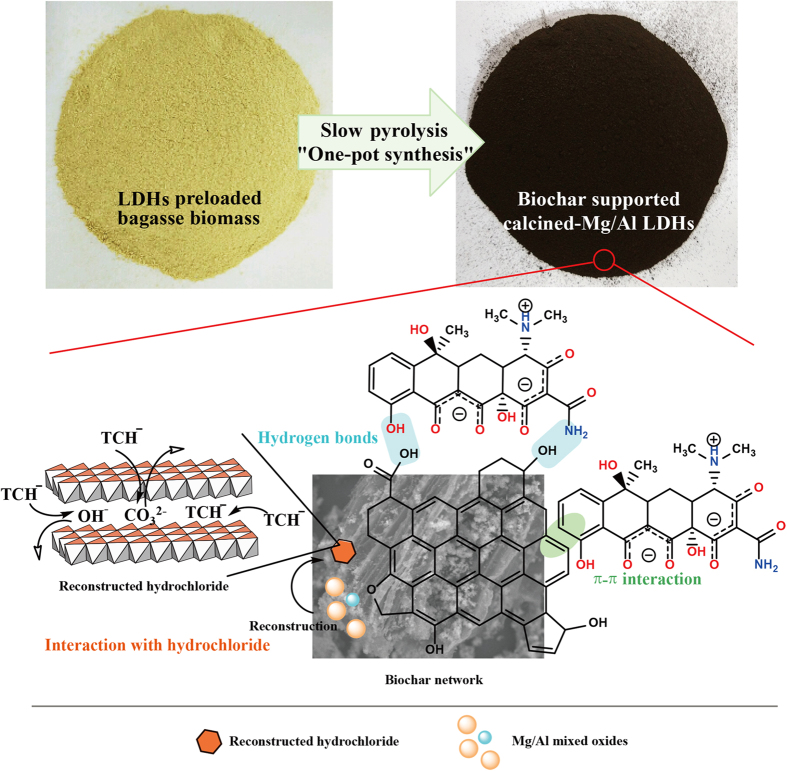
Schematic illustration of adsorption mechanisms of TC by CLDHs/BC.

As discussed in the effect of solution pH on TC adsorption, it is considered that the electrostatic interaction may not be the main adsorption force. Therefore, other complex and various kinds of interactions may be involved in the uptake of TC by CLDHs/BC. By combining the superior characteristic of calcined MgAl-LDH and biochar, CLDHs/BC can exert multi-effects on the adsorption of TC. Possible mechanisms of the adsorption of TC by the CLDHs/BC are proposed in Fig. 6. To verify these adsorption mechanism, the FT-IR spectrums (Fig. 1b) of CLDHs/BC and TC loaded CLDHs/BC (CLDHs/BC-TC) were investigated. After the adsorption of TC onto CLDHs/BC, the FT-IR spectrum exhibited many changes. The adsorption band of CLDHs/BC at 3430.8 cm^−1^ and 1056.8 cm^−1^ corresponding to the O−H stretching vibrations of hydrogen-bonded hydroxyl and the alkoxy C−O bending vibration slightly shifted to 3432.7 cm^−1^ and 1053.0 cm^−1^, respectively. This indicated that hydroxyl and alkoxy groups of CLDHs/BC played an important role in the adsorption process, which was ascribed to the formation of hydrogen bond by their interaction with hydroxyl and amino of TC^40^. It also can be seen that the adsorption peak at 1631.5 cm^−1^ belonged to C=C stretching vibrations for CLDHs/BC migrated to wavenumbers 1623.7 cm^−1^, which confirmed that π-π interaction between the benzene rings of TC and CLDHs/BC could be involved in TC adsorption^40^.

Furthermore, calcined Mg/Al-hydrotalcite have the ability to reconstruct their original layered structure after adsorption (“memory effect”) (El Gaini *et al*., 2009; Zhu *et al*., 2005), which can be expressed by the following equations (Lv *et al*., 2006b):


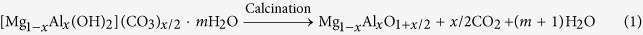



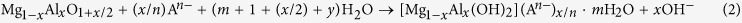


The characteristic FT-IR spectrum peaks of the M–O and O–M–O (M = Mg, Al) vibration at 611.3 cm^−1^, 540.0 cm^−1^, and 472.5 cm^−1^ shifted to 615.2 cm^−1^, 565.0 cm^−1^, and 468.6 cm^−1^ (due to M–O vibrations and M–O–H bending), respectively. This result confirmed the reconstruction of calcined Mg/Al hydrotalcite. The XRD pattern of CLDHs/BC-TC also exhibited the characteristic diffractions of hydrotalcite (indexed by the JCPDS X-ray powder diffraction file of No. 22-700.), indicating that the Mg/Al-hydrotalcite was successfully reconstructed after adsorption of TC. In the reconstruction process, anions are intercalated into the layered structure and OH^−^ ions are released simultaneously. The initial TC solution pH and final pH of the adsorption experiments are shown in Fig. 3b. As can be seen, the final pH significantly increased to 9.10–10.22. At these pH, TC mainly existed as anionic (TCH^−^ or TC^2−^) species (Fig. S3), and some can be intercalated into the interlayer space in the reconstructed Mg/Al-hydrotalcite^41^. In addition, the anion exchange between anionic TC and interlayer anion of hydrotalcite (such as carbonate and hydroxide ion) also played an important role in TC bonding.

### Comparison with other adsorbents and renewability evaluation

The comparisons of maximum adsorption capacities of CLDHs/BC of this study with other adsorbents reported previously for the adsorption of TC are listed in Table S5. As shown in the table, the TC adsorption capacity of CLDHs/BC is much higher than that of other adsorbents. In addition, it even showed higher adsorption ability for TC than some commercial activated carbons. Such comparison also suggests that CLDHs/BC may be an effective adsorbent for TC removal from contaminated water.

The regeneration of the exhaust adsorbent through desorption of the captured pollutants is an important process reflecting its renewability and economic value. The results indicated that the adsorption capacity decreased gradually with the increase of cycles (Fig. S5). NaOH exhibited about 70% desorption efficiency. The adsorption capacity of tetracycline decreased gradually, which might be attributed to the incomplete desorption in each cycle and the gradually weaker function of LDHs along with the increase of cycle. After five adsorption/desorption cycles, the adsorbed amount of TC onto the regenerated CLDHs/BC still remained equal to or higher than the pristine biochar and some reported adsorbents (Table S5). The results indicate that the CLDHs/BC could be a potential low cost and efficient adsorbent for TC removal due to its high adsorption capacity and excellent regeneration performance.

## Conclusions

In conclusion, biochar supported calcined-Mg/Al LDHs (CLDHs/BC) was synthesized using the one-pot fast pyrolysis reaction of Mg/Al LDHs preloaded onto bagasse biomass. Multiple characterizations of the resulting adsorbent illustrated that calcined-Mg/Al LDHs were successfully coated onto the pyrolyzed biochar and might provide more active sites. The prepared CLDHs/BC exhibited excellent performance for tetracycline removal. Multiple mechanisms are involved in the adsorption process including the interaction with LDHs, π–π interaction and hydrogen bond. CLDHs/BC is a high-performance and cost-effective material for antibiotics contaminated wastewater treatment. Further application of CLDHs/BC for other pharmaceuticals from wastewater and groundwater should be investigated.

## Materials and Methods

### Materials

Tetracycline hydrochloride (99%, w/w) was obtained from Hefei Bomei Biotechnology Co., Ltd., China. The details of tetracycline hydrochloride used in this study are shown in Table. S1. Molecular structures of different TC species as a function of pH are shown in Fig. S2. Stock solution (500 mg/L) was prepared in ultra-pure water (18.25 MΩ cm), and was used to acquire the initial concentrations of TC in batch sorption studies. The feedstock of biochar was bagasse, which was washed with distilled water and dried at 80 °C in a drying oven for 24 h. Then the dried bagasse were ground to powder and sieved through 60 mesh sieve (0.3 mm). The powdered bagasse was stored in airtight plastic containers for later use. All chemicals employed in the experiments were purchased at analytical reagent grade and without any further purification. All the solutions were prepared using ultra-pure water (18.25 MΩ cm).

### Preparation of biochar supported calcined-Mg/Al layered double hydroxides

Biochar supported calcined-Mg/Al layered double hydroxides was synthesized by pyrolyzing the Mg/Al layered double hydroxides pre-coated bagasse biomass. The Mg/Al layered double hydroxides was first coated onto bagasse biomass by a modified liquid-phase deposition method. Specifically, 20 g bagasse powder was impregnated with the Mg/Al solution (0.01 mol Al^3+^ and 0.03 mol Mg^2+^). The obtained suspension was reacted with a solution containing 0.4 mol NaOH and 0.125 mol Na_2_CO_3_ by adding them into a reactor simultaneously and dropwise. During the reaction process, the reactant was stirred and maintained the pH near 10. The resulting slurry was aged, then filtered and dried to obtain Mg/Al LDHs pre-coated bagasse biomass (LDHs/BM). The sample was then converted into biochar supported calcined-Mg/Al layered double hydroxides composite (CLDHs/BC) in a horizontal tube furnace (SK-1200 °C, Tianjin Zhonghuan Test Electrical Furnace Co., LTD, China) at 475 °C for 2 h. The detailed synthesis processes are provided in the Supplementary material section 1.

### Characterizations

The FTIR spectra (Nicolet 5700 Spectrometer, USA) of CLDHs/BC before and after adsorption were recorded in the range of 4000–400 cm^–1^. The X-ray diffraction (XRD) pattern was performed with a Bruker D8-Advance X-ray diffractometer (Bruker, German). The morphological structure of BC and CLDHs/BC was characterized by scanning electron microscope (SEM) (TM3000, Hitachi, Japan). Electron micrographs of the samples were taken by the transmission electron microscopy (TEM) on FEI Titan G2 60–300 microscope.

### Adsorption experiments

The effect of pH on the adsorption of TC was studied at room temperature (298 ± 0.5 K) in 100 mL Erlenmeyer flask containing 0.02 g of adsorbent and 50 mL TC solution. The initial TC solutions (100 mg/L) were adjusted ranging from 2.0 to 10.0 using solutions of NaOH and HCl. To investigate the effect of adsorbent dosage on the adsorption of TC, various adsorbent dosages from 0.01 g to 0.1 g were added to 50 mL of solution with TC initial concentration of 200 mg/L. The influence of salt ionic strength on the removal of TC by CLDHs/BC was studied with the sodium chloride and calcium chloride concentration varying from 0 to 0.1 M. The pH values of the TC solutions were adjusted to 6 and the flasks were shaken for 24 h at room temperature. The samples were then filtered through 0.45 μm filter and the concentrations of residual TC in the supernatant were determined by an UV-Vis spectrophotometer (UV-2550, SHIMADZU, Japan) at 357 nm^42^.

Adsorption kinetics were examined by mixing 0.02 g of adsorbent with 20 mL TC solution (20 mg/L) in 100 mL Erlenmeyer flask at room temperature (298 ± 0.5 K). The pH values of the TC solutions were adjusted to 6. The solutions were shaken at regular intervals, and the adsorbed TC concentrations were determined by the same method after filtration of the solution.

Adsorption isotherm and thermodynamic properties of TC onto CLDHs/BC was determined by batch sorption experiment under three different temperatures (298, 308, and 318 K) by mixing 0.02 g CLDHs/BC with 30 mL TC solutions of different concentrations ranging from 20 to 500 mg/L in the 100 mL Erlenmeyer flask. The pH values of the TC solutions were adjusted to 6. The Erlenmeyer flasks were shaken for 24 h. The samples were then withdrawn and filtered to determine adsorbed TC concentrations by the same method.

### Regeneration of used CLDHs/BC

The regeneration of CLDHs/BC was conducted by adding TC-loaded CLDHs/BC to 0.2 mol/L NaOH and the mixture was stirred at 298 K and 140 r/min for 24 h. After desorption, the regenerated CLDHs/BC was applied for next adsorption experiment. In the adsorption process, the regenerated CLDHs/BC was added into 50 mL TC solutions of 100 mg/L and shaken at 298 K for 24 h. The samples were then withdrawn and filtered to determine adsorbed TC concentrations.

## Additional Information

**How to cite this article**: Tan, X. *et al*. One-pot synthesis of carbon supported calcined-Mg/Al layered double hydroxides for antibiotic removal by slow pyrolysis of biomass waste. *Sci. Rep.* **6**, 39691; doi: 10.1038/srep39691 (2016).

**Publisher's note:** Springer Nature remains neutral with regard to jurisdictional claims in published maps and institutional affiliations.

## Supplementary Material

Supplementary Information

## Figures and Tables

**Table 1:** The model parameters and the corresponding correlation coefficient of kinetics models.

Kinetics	Parameters	
Pseudo-first-order	q_e,exp_ (mg/g)	17.70
	*q*_e,cal_ (mg/g)	16.76
	*K*_1_ (1/min)	0.047
	*R*^2^	0.702
Pseudo-second-order	q_e,exp_ (mg/g)	17.70
	*q*_e,cal_ (mg/g)	17.90
	*K*_2_ (g/mg min)	0.0044
	*R*^2^	0.999
Two-compartment model	*F*_fast_	0.58
	*F*_slow_	0.42
	*K*_fast_ (1/min)	70.96
	*K*_slow_ (1/min)	0.19
	*R*^2^	0.998

**Table 2:** The model parameters and the corresponding correlation coefficient of isotherm models.

Isotherms	Parameters	Temperature (K)		
		298	308	318
Langmuir	*q*_max_ (mg/g)	1002.38	1010.74	1118.12
	*K*_l_ (L/mg)	0.0025	0.0033	0.0042
	*R*^2^	0.996	0.997	0.997
	*R*_L_	0.43	0.36	0.31
Freundlich	1/*n*	0.86	0.85	0.84
	*K*_F_ (L/mg)	3.54	4.83	6.52
	*R*^2^	0.996	0.993	0.992
Tempkin	*K*_T_ (L/mg)	0.09	0.11	0.14
	*B*_T_	106.55	116.89	131.08
	*R*^2^	0.842	0.852	0.853
	*b*_T_ (J/mol)	24.81	21.91	18.90
BET	*K*_b_	127.13	169.80	212.69
	*q*_m_ (mg/g)	981.06	993.85	1103.05
	*R*^2^	0.996	0.997	0.998
