# Supplementary material for: One-pot synthesis of carbon supported calcined-Mg/Al layered double hydroxides for antibiotic removal by slow pyrolysis of biomass waste
Source: Sci Rep. 2016 Dec 21;6:39691. doi: 10.1038/srep39691 (PMC5175202; doi:10.1038/srep39691)
Supplement: Supplementary Information [file srep39691-s1.pdf]

**Supplementary Information for:**

# One-pot synthesis of carbon supported calcined-Mg/Al layered double hydroxides for antibiotic removal by slow pyrolysis of biomass waste

Xiaofei Tan [1,2], Shaobo Liu [3, 4] *, Yunguo Liu [1,2] *, Yanling Gu [1,2], Guangming Zeng [1,2], Xiaoxi Cai [1,2], ZhiLi Yan [1,2], Chunping Yang [1,2], Xinjiang Hu [1,2,5], Bo Chen [1,2]

[1] College of Environmental Science and Engineering, Hunan University, Changsha 410082, P.R. China.

[2] Key Laboratory of Environmental Biology and Pollution Control (Hunan University), Ministry of Education, Changsha 410082, P.R. China. [3] School of Metallurgy and Environment, Central South University, Changsha 410083, PR China. [4] School of Architecture and Art, Central South University, Changsha 410083, PR China. [5] College of Environmental Science and Engineering Research, Central South University of Forestry and Technology, Changsha 410004, PR China. Correspondence and requests for materials should be addressed to Y.L. (email: hnuliuyunguo@gmail.com; liuyunguo@hnu.edu.cn) and S.L. (email: liushaobo23@aliyun.com)

**17 pages, 2 sections, 5 tables, and 5 figures.**

**1. Preparation of biochar supported calcined-Mg/Al layered double hydroxides**

Biochar supported calcined-Mg/Al layered double hydroxides was synthesized by pyrolyzing the Mg/Al layered double hydroxides pre-coated bagasse biomass. MgAl layered double hydroxides was first synthesized and coated on the biomass feedstock (powdered bagasse) before pyrolysis by a modified liquid-phase deposition method. $AlCl_3 \cdot 6H_2O$ (2.4 g, 0.01 mol) and $MgCl_2 \cdot 6H_2O$ (6.1 g, 0.03 mol) were dissolved in deionized water. 20 g of the bagasse powder was impregnated with the Mg/Al solution, and then the mixture was shaken at 130 r/min for 24 h. A second solution containing NaOH (16.0 g, 0.4 mol) and $Na_2CO_3$ (13.25 g, 0.125 mol) in 500 mL deionized water was prepared. The two solutions (Mg/Al solution and the mixture of $Na_2CO_3$ and NaOH) were added into a three-necked flask, simultaneously and dropwise, while stirring with rotor speed of 500 r/min and maintaining the pH near 10. The pH was maintained constant by the simultaneous addition of the mixture of $Na_2CO_3$ and NaOH. The resulting slurry was aged at 100 ℃ for 24 h. The final precipitate was filtered and dried at 80 ℃ for 36 h to obtain Mg/Al LDHs pre-coated bagasse biomass (LDHs/BM). A horizontal tube furnace (SK-1200 ℃, Tianjin Zhonghuan Test Electrical Furnace Co., LTD, China) was used to convert these samples into biochar. MgAl LDHs-coated biomass powder was filled in a quartz boat and loaded in the quartz tube furnace. Both ends of the quartz tube were sealed and connected with hoses. The chamber of tube furnace was replenished with $N_2$ (400 mL/min) from one side to keep the inert atmosphere along with the pyrolysis process and the volatile products during biomass pyrolysis are collected from another side. The temperature was programmed to rise up to 500 ℃ at a rate of 7 ℃/min and held

at the peak temperature for 2 h before cooling to room temperature. The sample was then converted into biochar supported calcined-Mg/Al layered double hydroxides composite (CLDHs/BC). The pristine biochar (BC) without LDHs treatment was also produced under the same pyrolysis condition.

## 2. Mathematical Models

### 2.1. Adsorption kinetics

The pseudo-first-order, pseudo-second-order and intra-particle diffusion models were applied to simulate the experimental kinetic data. The equations are generally expressed as follows [1]:

$$\log\left(q_e - q_t\right) = \log q_e - k_1 / 2.303t \quad (1)$$

where $q_e$ (mg/g) and $q_t$ (mg/g) represented the sorption amount of TC at equilibrium and at time $t$ (min); $k_1$ (1/min) is the pseudo-first-order reacted rate constant. $k_1$ and $q_e$ is calculated from the slope and intercept of the plots of log ($q_e - q_t$) versus $t$, respectively.

$$\frac{t}{q_t} = \frac{1}{k_2 q_t^2} + \frac{t}{q_e} \quad (2)$$

where $q_e$ (mg/g) and $q_t$ (mg/g) represented the sorption amount of TC at equilibrium and at time $t$ (min); $k_2$ (g/mg.min) is the rate constant of pseudo-second-order adsorption determined by the plots of $t/q_t$ versus $t$.

$$q_t = k_{id} t^{1/2} + c_i \quad (3)$$

$k_{id}$ is the intra-particle diffusion rate constant (mg/g ·min$^{0.5}$), and $c_i$ is the intercept related to the thickness of the boundary layer. The larger the intercept, the greater the

contribution of the surface sorption in the rate controlling. According to this model, if the value of $c_i$ was zero, it means that the rate of adsorption is controlled by intra-particle diffusion for the entire adsorption period. Also, $q_t$ versus $t^{1/2}$ should be linear if intra-particle diffusion was involved in the adsorption process.

Furthermore, the time-courses of TC sorption onto ramie biochars were analyzed with a two-compartment and first-order dynamics model, which can be presented as follows [2]:

$$\frac{q_t}{q_{t=\infty}} = F_{fast}(1-e^{-t/K_{fast}}) + F_{slow}(1-e^{-t/K_{slow}}) \tag{4}$$

where $q_t$ (mg/g) and $q_{t=\infty}$ (mg/g) are the amounts of adsorbed TC on the CLDHs/BC at time t and equilibrium, respectively. Parameters of $F_{fast}$ and $F_{slow}$ are the mass fractions of ‘‘fast” and ‘‘slow” compartments, respectively, and $F_{fast} + F_{slow} = 1$. Parameters of $K_{fast}$ (1/min) and $K_{slow}$ (1/min) are the first-order rate constants for transfer into ‘‘fast” and ‘‘slow” compartments of the CLDHs/BC by two-compartment model.

To gain insights into the actual rate-controlling step involved in the overall TC sorption process, the adsorption kinetic data were further analyzed using the Boyd kinetic model, which is expressed as follows [3, 4]:

$$F = 1 - \frac{6}{\pi^2}\exp(-B_t) \tag{5}$$

where $F$ is the fraction of adsorbate adsorbed at different time $t$. $B_t$ is a mathematical function of $F$, which is given by:

$$F = \frac{q_t}{q_e} \tag{6}$$

where $q_t$ and $q_e$ are the adsorption quantity at time $t$ and equilibrium, respectively. The kinetic expression Eq. (4) can be represented as:

$$B_t = -0.4977 - \ln(1-F) \tag{7}$$

2.2. Adsorption isotherms

The adsorption equilibrium isotherms was studied using four adsorption isothermal models including Langmuir, Freundlich, Tempkin and BET models to fit the experimental data. The equations of these adsorption models are expressed, respectively, by the following equations [1]:

$$\frac{C_e}{q_e} = \frac{C_e}{q_{max}} + \frac{1}{q_{max}K_L} \tag{8}$$

where $q_e$ is the amount of the TC adsorbed (mg/g), $C_e$ is the equilibrium concentration of solution (mg/L), $q_{max}$ is the maximum adsorption capacity (mg/g), and $K_L$ (L/mg) is the Langmuir constant related to the affinity, which is used to indicate whether the adsorption equilibrium is favorable ($0< K_l <1$) or unfavorable ($K_l >1$).

$$\ln q_e = \ln K_F + \frac{1}{n}\ln C_e \tag{9}$$

where $q_e$ is the amount of the TC adsorbed (mg/g), $C_e$ is the equilibrium concentration of solution (mg/L). $K_F$ (L/mg) and $n$ are the Freundlich constants, which indicate the adsorption capacity and intensity, respectively.

$$q_e = B_T \ln K_T + B_T \ln C_e \tag{10}$$

where $q_e$ is the amount of the TC adsorbed (mg/g), $C_e$ is the equilibrium concentration of solution (mg/L). $B_T = RT/b_T$ and $b_T$ (J/mol) is the Temkin constant related to the heat of adsorption, $R$ is the universal gas constant (8.314 J/mol.K) and $T$ is the temperature (K); $K_T$ (L/mg) is the maximum binding energy constant.

$$\frac{C_e}{(C_s - C_e)q_e} = \frac{1}{K_b q_m} + \frac{C_e(K_b - 1)}{K_b q_m C_s} \tag{11}$$

where $C_e$ is the equilibrium concentration of adsorbate in solution (mg/L), $C_s$ is saturation concentration of solute (mg/L), $q_e$ is the final amount of adsorbate adsorbed onto adsorbent (mg/g), $q_m$ is the amount of solute adsorbed in forming a complete monolayer (mg/g), $K_b$ is the BET constant.

2.3. Thermodynamic analysis

Thermodynamic analysis was taken to gain further insights into sorption process and mechanisms. The thermodynamic data, such as Gibbs free energy $\Delta G^0$, enthalpy $\Delta H^0$, entropy $\Delta S^0$, can be calculated using the following equations [5]:

$$\Delta G^0 = -RTK^0 \tag{12}$$

$$\ln k^0 = \frac{\Delta H^0}{RT} + \frac{\Delta S^0}{R} \tag{13}$$

where $R$ is the gas constant 8.314 J/mol K, $T$ (K) is the absolute temperature in Kelvin; $K^0$ could be calculated by plotting $\ln(q_e/c_e)$ versus $c_e$ and extrapolating $c_e$ to zero. From the linear plot of ln $K^0$ versus $1/T$, the values of $\Delta H^0$ (kJ/mol) and $\Delta S^0$ (kJ/mol K) could be obtained from the slope and intercept.

**Table S1.** Details of the tetracycline hydrochloride used in this study

| Tetracycline hydrochloride | |
|---|---|
| IUPAC name | (4S,4aS,5aS,6S,12aR)-4-(dimethylamino)-1,6,10,11,12a-penta hydroxy-6-methyl-3,12-dioxo-4,4a,5,5a-tetrahydrotetracene-2-carboxamide;hydrochloride |
| Commercial name | Tetracycline hydrochloride |
| CAS number | 64-75-5 |
| Appearance | Yellow crystalline powder |
| Molecular Formula | $C_{22}H_{25}ClN_2O_8$ |
| Molecular weight | 480.9 g/mol |
| Melting point | 220-223 °C |
| Water Solubility | 50 g/L |
| $pK_a$ | $pK_{a1}$ = 3.3, $pK_{a2}$ = 7.7, $pK_{a3}$ = 9.7 |
| Molecular structure | 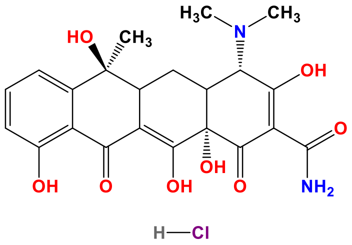 |

**Table S2.** Intra-particle diffusion parameters for the adsorption of TC by CLDHs/BC.

| | $k_{id}$ (mg/g·min$^{0.5}$) | $c_i$ | $R_i^2$ |
|---|---|---|---|
| Section1 | 0.83 | 6.45 | 0.991 |
| Section2 | 0.13 | 15.41 | 0.925 |
| Section3 | 0.0059 | 17.46 | 0.922 |

**Table S3.** Boyd plot parameters for the adsorption of TC by CLDHs/BC.

| | $k_{id}$ | $c_i$ | $R_i^2$ |
|---|---|---|---|
| Section1 | 0.014 | 0.045 | 0.992 |
| Section2 | 0.00044 | 3.35 | 0.890 |

**Table S4.** Thermodynamic parameters for TC adsorption on CLDHs/BC.

| T (K) | Δ*G*° (kJ/mol) | Δ*H*° (kJ/mol) | Δ*S*° (J/mol K) | $R^2$ |
|---|---|---|---|---|
| 298 | -2.23 | 20.86 | 69.13 | 0.998 |
| 308 | -3.06 | | | |
| 318 | -4.03 | | | |

**Table S5.** Maximum adsorption capacities of various adsorbents for TC.

| Adsorbent | Temperature (K) | $q_{max}$ (mg/g) | References |
|---|---|---|---|
| Carbon supported nano-Mg/Al hydrotalcite | 298 | 1002.38 | In this study |
| NaOH-activated sludge | 298 | 672.0 | [6] |
| Commercial granular activated carbons Merck (M) | 298 | 471.1 | [7] |
| NaOH-activated carbon | | 455.8 | [8] |
| Commercial granular activated carbons Sorbo Norit (S) | 298 | 375.4 | [7] |
| Graphene oxide | 298 | 313.l48 | [9] |
| Multi-walled carbon nanotubes with 3.2% oxygen contents | 298 | 269.25 | [10] |
| Magnetic biochar | 298 | 94.2 | [11] |
| Alkali biochar | 303 | 58.8 | [12] |
| Ferric-activated sludge-based adsorbent | 298 | 54.53 | [13] |
| Magnetic activated carbon | 298 | 45.3 | [11] |

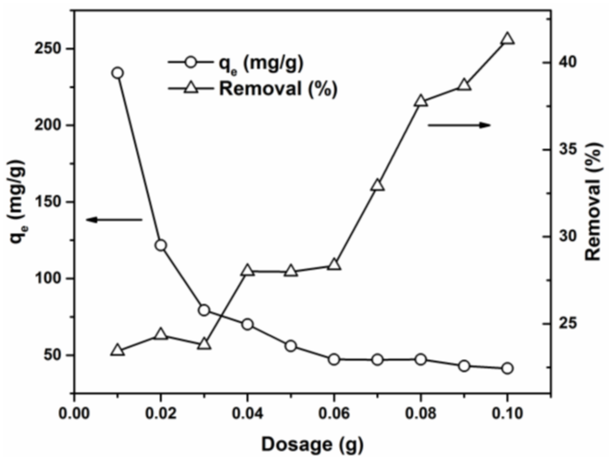


**Fig. S1.** Effects of the CLDHs/BC dosage on the adsorption of TC.

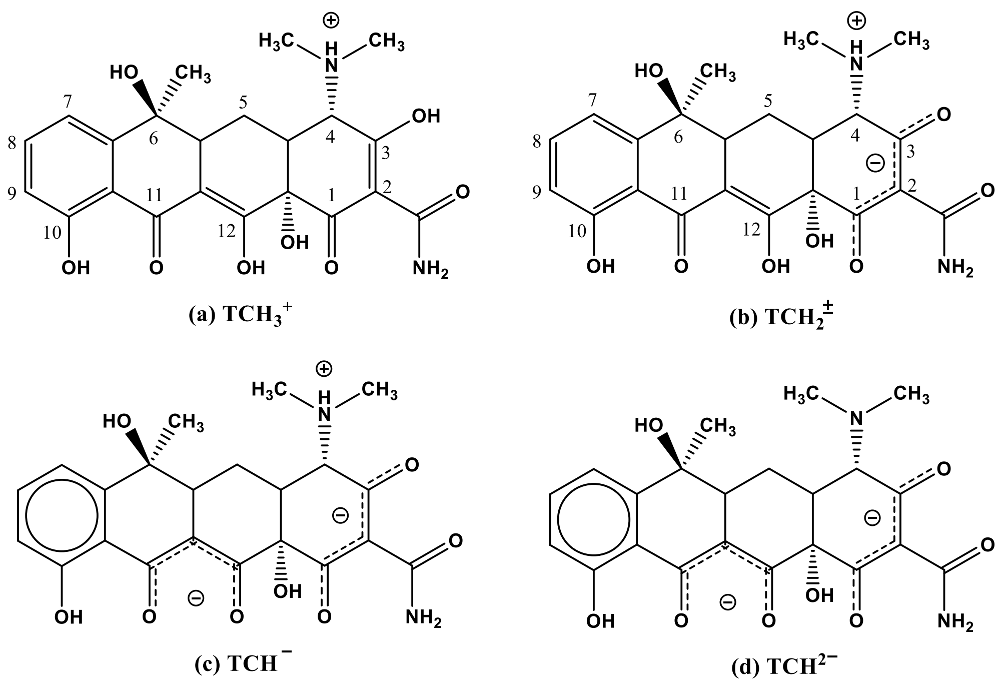


**Fig. S2.** Molecular structures of different TC species as a function of pH.

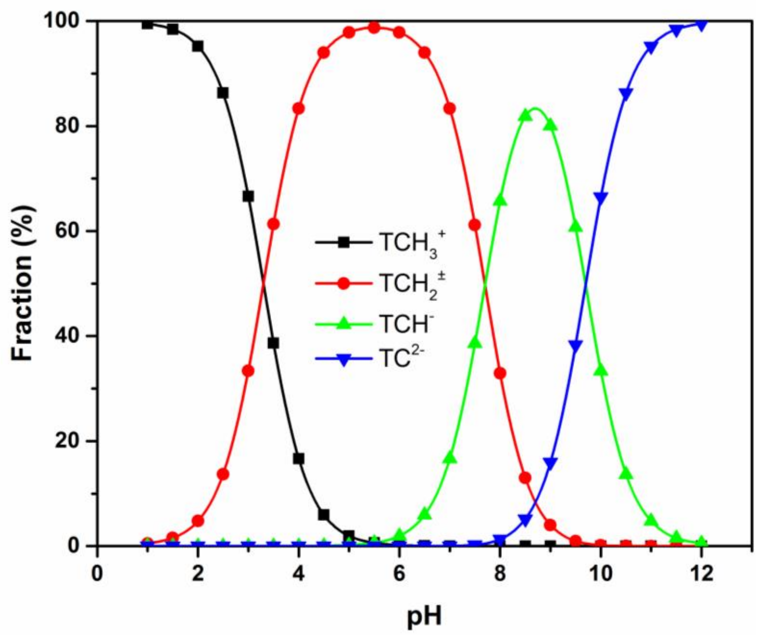


**Fig. S3.** The speciation of TC under different pH conditions.

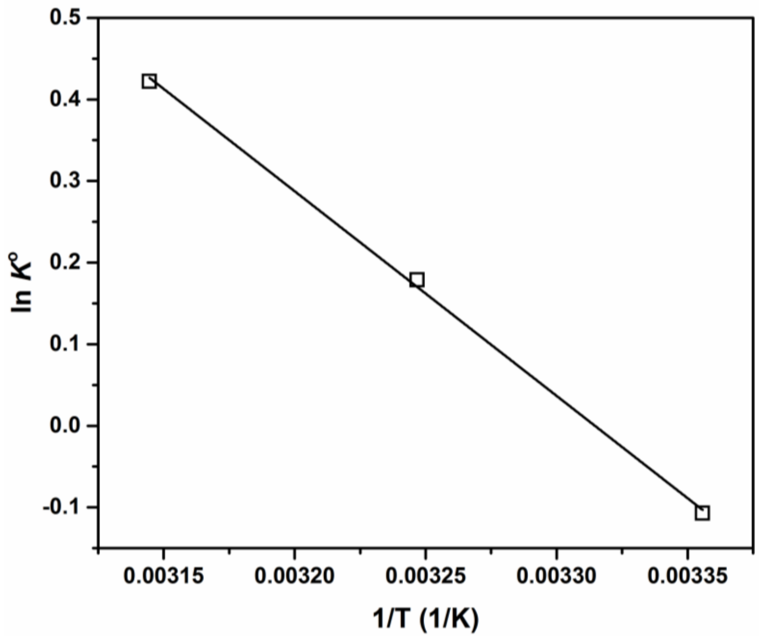


**Fig. S4.** Linear plot of ln *K*° versus 1/T for the adsorption of TC on CLDHs/BC at 298, 308, and 318 K.

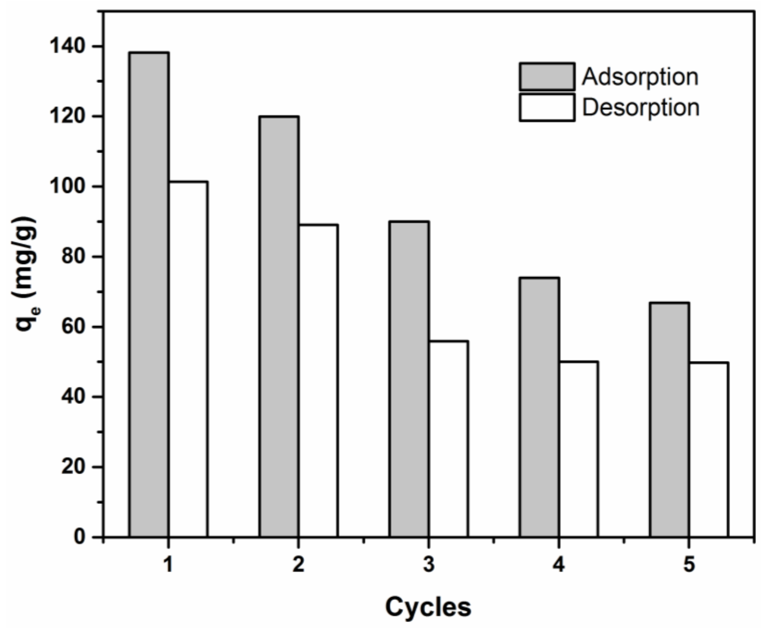


**Fig. S5.** Five adsorption-desorption cycles of CLDHs/BC for TC removal.

## References


1. Hu, X. J. *et al.* Adsorption of chromium (VI) by ethylenediamine-modified cross-linked magnetic chitosan resin: isotherms, kinetics and thermodynamics. *J. Hazard. Mater.* **185**, 306-314 (2011).
2. Zhou, L. *et al.* Investigation of the adsorption-reduction mechanisms of hexavalent chromium by ramie biochars of different pyrolytic temperatures. *Bioresour. Technol.* **218**, 351-359 (2016).
3. Ai, L. H. *et al.* Adsorption of methyl orange from aqueous solution on hydrothermal synthesized Mg–Al layered double hydroxide. *J. Chem. Eng. Data* **56**, 4217-4225 (2011).
4. Boyd, G. *et al.* The exchange adsorption of ions from aqueous solutions by organic zeolites. II. Kinetics1. *J. Am. Chem. Soc.* **69**, 2836-2848 (1947).
5. Sun, Z. C. *et al.* Fast adsorption of $Cd^{2+}$ and $Pb^{2+}$ by EGTA dianhydride (EGTAD) modified ramie fiber. *J. Colloid Interface Sci.* **434**, 152-158 (2014).
6. Ocampo-Pérez, R. *et al.* Kinetic study of tetracycline adsorption on sludge-derived adsorbents in aqueous phase. *Chem. Eng. J.* **213**, 88-96 (2012).
7. Ocampo-Pérez, R. *et al.* Modeling adsorption rate of tetracyclines on activated carbons from aqueous phase. *Chem. Eng. Res. Des.* **104**, 579-588 (2015).
8. Martins, A. C. *et al.* Removal of tetracycline by NaOH-activated carbon produced from macadamia nut shells: Kinetic and equilibrium studies. *Chem. Eng. J.* **260**, 291-299 (2015).
9. Gao, Y. *et al.* Adsorption and removal of tetracycline antibiotics from aqueous solution by graphene oxide. *J. Colloid Interface Sci.* **368**, 540-546 (2012).
10. Yu, F. *et al.* Adsorption of tetracycline from aqueous solutions onto multi-walled carbon nanotubes with different oxygen contents. *Sci. Rep.* **4**, 5326 (2014).
11. Shan, D. *et al.* Preparation of ultrafine magnetic biochar and activated carbon for pharmaceutical adsorption and subsequent degradation by ball milling. *J. Hazard. Mater.* **305**, 156-163 (2016).
12. Liu, P. *et al.* Modification of bio-char derived from fast pyrolysis of biomass and its application in removal of tetracycline from aqueous solution. *Bioresour. Technol.* **121**, 235-240 (2012).
13. Yang, X. *et al.* Preparation of ferric-activated sludge-based adsorbent from biological sludge for tetracycline removal. *Bioresour. Technol.* **211**, 566-573 (2016).
